# Recent Advances in Metalloproteomics

**DOI:** 10.3390/biom14010104

**Published:** 2024-01-13

**Authors:** James P. C. Coverdale, Sirilata Polepalli, Marco A. Z. Arruda, Ana B. Santos da Silva, Alan J. Stewart, Claudia A. Blindauer

**Affiliations:** 1 School of Pharmacy, Institute of Clinical Sciences, University of Birmingham, Edgbaston B15 2TT, UK; j.p.coverdale@bham.ac.uk; 2 Department of Chemistry, University of Warwick, Coventry CV4 7AL, UK; sirilata.polepalli@warwick.ac.uk; 3 Institute of Chemistry, Department of Analytical Chemistry, Universidade Estadual de Campinas, Campinas 13083-970, Brazil; zezzi@unicamp.br (M.A.Z.A.); ana.bs.silva@unesp.br (A.B.S.d.S.); 4 School of Medicine, University of St. Andrews, St Andrews KY16 9TF, UK

**Keywords:** metalloproteome, essential metals, xenobiotic metals, metallodrugs, ligand exchange kinetics

## Abstract

Interactions between proteins and metal ions and their complexes are important in many areas of the life sciences, including physiology, medicine, and toxicology. Despite the involvement of essential elements in all major processes necessary for sustaining life, metalloproteomes remain ill-defined. This is not only owing to the complexity of metalloproteomes, but also to the non-covalent character of the complexes that most essential metals form, which complicates analysis. Similar issues may also be encountered for some toxic metals. The review discusses recently developed approaches and current challenges for the study of interactions involving entire (sub-)proteomes with such labile metal ions. In the second part, transition metals from the fourth and fifth periods are examined, most of which are xenobiotic and also tend to form more stable and/or inert complexes. A large research area in this respect concerns metallodrug–protein interactions. Particular attention is paid to separation approaches, as these need to be adapted to the reactivity of the metal under consideration.

## 1. Introduction

Although the metal content of a typical organism is less than 1%, it is fair to say that life would be impossible without metal ions: not only are they required for the function of about half of all protein-based enzymes [1], but they are also required for many reactions involving the backbone of nucleic acids [2] and are involved in cellular signalling [3]. Metal ions and their complexes can also be used for diagnostic and therapeutic purposes [4]. These include Pt and Ru-anticancer complexes (e.g., cisplatin and KP1019, respectively) and Au-antiarthritic complexes (e.g., auranofin). Most often, their biological activity involves the interaction of xenobiotic metals (or their complexes) with either proteins or nucleic acids. Finally, the wrong metal, whether xenobiotic or essential, in the wrong place can lead to damage, illness, and death [5].

In recognition of the importance of metal ion–protein interactions, a steady increase in studies involving “Metalloproteomics” has taken place in the past decade [6,7,8,9,10,11,12,13,14,15,16], also fuelled by major developments in analytical instrumentation. Before we continue with reviewing progress in this area, it is necessary to define what we mean by “Metalloproteomics”. In the broadest sense, it is a sub-discipline of “Metallomics” [17,18,19,20] and may be seen to encompass studies aimed at understanding interactions between metal ions and proteins—i.e., a large proportion of Inorganic Biochemistry. We believe that this definition is, perhaps, too broad, and propose that metalloproteomics is inherently related to chemical speciation [20,21,22], either in complex systems (as the “-omics” suffix would suggest) or in conjunction with single proteins where more than one metallospecies may be present. As such, we also propose that metalloproteomics is a sub-discipline of “Speciomics”, a concept we have defined recently [23].

In terms of methodology, although speciation information can be derived from a multitude of techniques, including a range of spectroscopies such as those based on X-ray absorption [21,24], a large proportion of metalloproteomics involves mass spectrometry (MS; for a list of abbreviations, see Abbreviations)-based techniques, both molecular and inorganic (or “elemental”). It is clear that the emergence of metalloproteomics has only been possible because of the immense progress that has been made in MS instrumentation and the associated increase in sensitivity for the detection of both organic molecules including proteins (typically by Electrospray Ionisation (ESI-) or Matrix-Assisted Laser Desorption Ionisation (MALDI-) MS) and inorganic elements (typically through Inductively-Coupled Plasma Mass Spectrometry (ICP-MS) [25])—and their complexes. We also observe that the term “Proteomics” in itself, even when restricted to MS-based methodology, does not only encompass the study of entire proteomes or sub-proteomes, but also is found referring to experimental approaches applied to single proteins—typically involving tandem-MS techniques. This review, however, focuses on the analysis of complex mixtures.

For complex systems containing multiple proteins, separation techniques are, of course, indispensable [26,27], and, perhaps, this remains the area with the largest potential for further development [28]. This requires an understanding of both separation science and coordination chemistry. In its early days, metalloproteomics was dominated—with few exceptions [29]—by pioneering analytical chemists with experience in metal ion speciation [21,30,31,32,33]. These early studies also testify to an intrinsic understanding of how different metal ions behave [30], although this has rarely been stated explicitly. We, therefore, believe that the coordination chemistry aspects of metalloproteomics are not necessarily appreciated widely and aim to address this to some extent in the present review.

**Figure 1:**
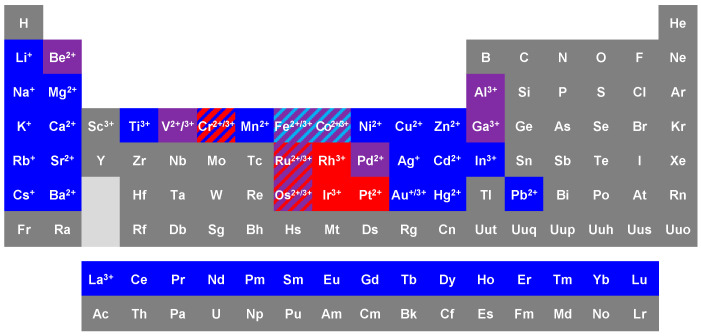
Metal ions may be classified into labile and inert according to the water exchange rates of their aqua complexes [34]. Water exchange rate constants at 298 K (*k*(H_2_O)) vary over more than 18 orders of magnitude. Labile metal ions (*k*(H_2_O) = 10^10^–10^4^ s^−1^) are shown in blue, inert metal ions (*k*(H_2_O) = 10^−3^–10^−10^ s^−1^) in red, and intermediate metal ions in purple. Hatched boxes indicate faster exchange rates for M^2+^ and slower exchange rates for M^3+^ ions. Grey boxes are shown for non-metals, metalloids, and metals for which oxidation states between I and III are rare or for which the respective data were not available.

First and foremost, we emphasise that it is the thermodynamic and kinetic properties of the complexes under study that determine which analytical approaches (both separation and MS-based) have the best potential to yield meaningful results. These properties ultimately determine the likelihood of the complexes dissociating during analysis, and which experimental conditions may minimise this. We have, therefore, divided our review of recent work into separate sections for labile and inert complexes. “Labile” and “inert” are, of course, kinetic terms and ultimately refer to how fast the bonds between the metal ion and its ligands are broken (and formed) [34]. These kinetic properties are defined by the metal ion (Figure 1) as well as by its ligand(s).

The bonds between metal ions and their ligands are, in terms of their origin, so-called “dative bonds”, which means that they typically come about by a ligand donating a lone pair to a usually positively-charged metal ion (at least for classical coordination complexes that prevail in aqueous solution). As such, electrostatic interactions are important contributors to bond strength as well as to kinetic properties, with a higher charge density being associated with stronger bonds and slower kinetics. Nonetheless, depending on the metal ion, the resulting bonds can have significant covalent character, which impacts on their strength as well as on the kinetics of breaking them. In a first approximation, the bonds in most biologically relevant complexes involving metal ions from the s-, p-, and f-block and the 3d row are dominated by ionic contributions and are labile, with ligand-field effects additionally affecting the d-block metals (Figure 1). For instance, owing to the d^3^ configuration, Cr^3+^ in octahedral coordination geometry is inert. In contrast, the 4d and 5d M^2+^ or M^3+^ metal ions in groups 8–10 form complexes with significant covalent character and are much more inert than their 3d congeners. Thus, Section 2 focuses on labile complexes and comprises many examples of complexes with essential metals (many of which are 3d), but also with metals of interest in the context of clinical diagnostics (lanthanides). We also include several studies on the 4d and 5d metal ions Ag(I), Cd(II), and Hg(II) in this section. According to the data compiled in Figure 1, they form labile complexes, owing to a low charge density and full d-shells. However, because of their high thermodynamic stability (especially for Hg(II)), their complexes may typically behave more robustly than those of 3d row metal ions and may, thus, tolerate harsher experimental conditions. Moreover, Ag(I) and Hg(II) tend to form only one or two bonds with proteins, so their interactions may also be less affected by protein unfolding. In turn, Section 3 focuses on inert complexes, all of which pertain to xenobiotic metals, often in the context of metallodrugs such as cis-platin, its derivatives, and Ru-based complexes with anti-cancer activity. Finally, therapeutic gold complexes are also briefly considered in a separate section: Au(I)/(III) are not inert, but the bonds they form with proteins may be rather strong, which may facilitate their metalloproteomic analysis. We note that, although essential, selenium tends to occur in inert form in selenoproteins. However, as selenium behaves like a non-metal in these compounds (and forms stable covalent bonds), we do not include metalloproteomic studies on selenoproteins in this review. We also do not include studies where “standard” proteomics techniques are employed to study any impacts of changes in the levels of metal ions, as these do not involve the explicit study of actual metal–protein complexes. Furthermore, we do not include theoretical approaches (i.e., bioinformatics; see, e.g., [35]), although it may be noted that the advent of AlphaFold and its descendants has already led to a further expansion of “the metalloproteome” [36].

## 2. Labile Complexes

### 2.1. General Considerations

**Figure 2:**
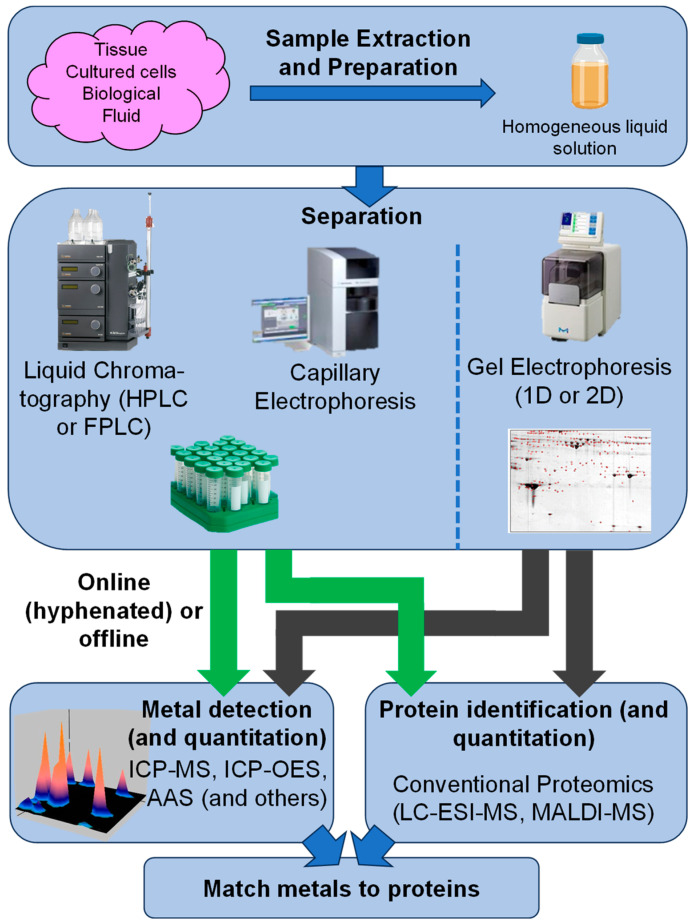
Typical workflows for metalloproteomics and associated techniques. Metal–protein associations need to be preserved during sample extraction and preparation as well as during each separation step.

As seen in Figure 1, the majority of metal ions form labile complexes, irrespective of whether these are held together by strong or weak bonds. Bond strength is also important, but most essential metals form bonds of only moderate strength. These properties have several consequences, both in terms of how the metallation of metal-requiring proteins occurs in vivo [37] and how samples for metalloproteomic studies need to be handled [28]. Typical workflows for such studies are illustrated in Figure 2. The following considerations apply to sample extraction and preparation as well as to the separation of complex mixtures but are also valid for the purification of single proteins.

In proteins where the metal ion is bound directly by amino acid side-chains (i.e., not immobilised by chelators such as protoporphyrins), it is often possible to remove the metal ion directly through a reaction with a chelator such as EDTA (2,2′,2′′,2′′′-(ethane-1,2-diyldinitrilo)tetraacetic acid), TPEN (*N*^1^,*N*^1^,*N*^2^,*N*^2^-tetrakis[(pyridin-2-yl)methyl]ethane-1,2-diamine), or bipyridine if the affinity and/or concentration of the latter exceeds that of the protein, and the metal site is accessible. These conditions are met by many of the complexes that occur in the context of metal ion trafficking and signalling: in the case of proteins involved in trafficking, there is a requirement for the metal ion to move from protein to protein, either directly through protein–protein interactions, or indirectly through dissociation and association, whilst in the case of signalling, once the regulatory or signalling event has taken place, the metal–protein complex needs to be deactivated again, typically through dissociation (e.g., as a consequence of a drop in metal ion concentration). In contrast, in at least some enzymes, the metal ion is often buried and cannot be removed easily (i.e., it is apparently inert)—but only as long as the protein remains folded; if the protein becomes denatured (unfolded), this will, in most cases, expose and/or destroy the metal-binding site, rendering the complex labile and, hence, also prone to dissociation. This generates the first mandate for metalloproteomics of complexes with labile metal ions—that experimental conditions must ensure that proteins remain folded (Table 1).

Furthermore, any liganding moieties (i.e., sites with a lone pair) have affinity for both metal ions and protons, which means that the latter can, in principle, compete for any metal binding site. This generates the second mandate: experimental conditions typically cannot involve low pH. Thus, the choice of buffers for extraction and separation is critical. In a first approximation, buffers should ideally mimic the properties and composition of the original environment (pH, ionic strength, and/or metal concentrations). Mobile phases for separation are discussed in more detail below and are specified for most selected examples. It is important to note that both of these mandates are violated by several standard approaches used in classical proteomics, which often involve working under acidic conditions that will both unfold proteins and dissociate complexes with labile essential metal ions. Moreover, enzymatic digestion, another common step in bottom-up proteomics, will further destroy metal sites.

A third consideration concerns redox chemistry, relevant for redox-active metals (first and foremost, iron and copper) as well as redox-active amino acid sidechains (in particular, cysteine). The cytosol is a reducing environment; hence, exposure to air during the preparation of cell lysates or tissue extracts introduces considerable perturbation of the redox state. Depending on the proteins of interest, working anaerobically (i.e., under nitrogen or argon) is necessary (see, e.g., [38,39,40]). This issue is less problematic for extracellular fluids because here, the environment is not reducing, and, hence, exposure to air generates fewer problems.

Fourthly, and this is especially important for attempts to determine cellular metalloproteomes, the preparation of cell lysates or tissue extracts is highly likely to lead to the redistribution of metal ions [18]. This is owing to the removal of membrane barriers between cellular compartments, the chemical composition of which can be quite different. This might be somewhat alleviated by the preparation of subcellular fractions, but care should also be taken here to ensure that the reagents neither denature proteins nor dissociate metal–protein complexes (via a low pH or the presence of chelators such as EDTA, with the latter being a regrettably frequent component of commercial kits [41]). In addition, since sample amounts are often small, contamination can become an issue. Therefore, it is highly advisable to employ trace-metal clean methodology, including pre-treating any reagent solutions with chelating agents or resins and avoiding glassware and certain types of plasticware to prevent the introduction of metals that can leach from such materials. Advantages of rapid sample throughput have been emphasised [42], and it is preferable to process samples immediately. If immediate analysis is not possible, samples will need to be stored under conditions that minimise the degradation of any biomolecules present (typically through the rapid freezing of samples and storage at −80 °C), as degraded proteins are likely to lose their bound metals.

Even when all the above considerations are adhered to, it is still the case that we are not in a position to determine full native metalloproteomes [18]. Apart from the fact that even mere “full proteomes” of cells or organisms remain elusive [43], the persistent major challenge in the case of metalloproteomes concerns the difficulty in matching metals to proteins. This is owing to (i) the sheer number of proteins in a given proteome, (ii) limitations in the resolution of any fractionation and separation techniques, and (iii) limitations to the sensitivity of both molecular and inorganic mass spectrometries. In theory, one could attempt to purify every single protein, and then identify which metal(s) is/are bound, but even if complications such as metal redistribution and protein degradation were not present, this is simply not practical. However, many recent studies have shown that the major binding partners for a metal ion of interest can be identified (see Section 2.5 and Section 3).

### 2.2. Separation Techniques to Analyse Complex Metalloprotein Mixtures

Separation science plays an important role in metalloproteomics, with (multidimensional) liquid chromatographies (LC; in particular, size-exclusion (SEC) and anion exchange (AEX) chromatography (either in HPLC or FPLC format [26])) and electrophoresis (either gel (GE) [44] or capillary electrophoresis (CE) [45]) being most commonly employed.

The importance of choosing an appropriate mobile phase cannot be overstated. This has been recognised in a study employing SEC-ICP-OES to profile the Fe-, Zn-, and Cu-proteomes from rabbit plasma [46]. Perhaps surprisingly, MOPS (3-(Morpholin-4-yl)propane-1-sulfonic acid) and HEPES (2-[4-(2-Hydroxyethyl)piperazin-1-yl]ethane-1-sulfonic acid) buffers were highlighted as problematic for the examination of Fe(III)- and Zn-protein complexes. Tris(hydroxymethyl)aminomethane (Tris)-HCl was also deemed unsuitable for Zn-protein complexes in this system, whereas phosphate-buffered saline was identified as preferable. However, phosphate-based buffers have other drawbacks, including limited compatibility with ICP-MS and the possibility of extracting and precipitating a range of metal ions from their native binding partners [47]. The latter study explored the merit of different SEC mobile phases applied to various sample types including brain homogenate, Cu/Zn-superoxide dismutase (SOD1), and a molecular weight standard mix containing ferritin, caeruloplasmin, cytochrome c, vitamin B12, and thyroglobulin. Fractions were analysed using ICP-MS for Fe, Cu, and Zn. When protein standards were analysed, HEPES and MOPS caused significant matrix suppression, whilst ammonium nitrate (NH_4_NO_3_; 20–100 mM) and Tris-HCl gave higher sensitivity. However, when brain homogenates were analysed, NH_4_NO_3_ had the disadvantage of leading to the loss of Zn and Fe from proteins and their retention on the SEC column (Agilent Bio SEC-3), as well as the introduction of additional Cu into apo-SOD1. Similarly, Tris-HCl also increased the amount of Zn recovered in eluates. Due to the latter observations, the study also investigated the impact of the stationary phase. Silica- and dextran-based phases behaved similarly in terms of the transfer of Zn from proteins to stationary phase, but the silica-based column donated more Cu to apo-SOD1 than the dextran-based phase. In summary, the use of Good’s buffers (MOPS, HEPES, and PIPES) was strongly discouraged, whilst NH_4_NO_3_ (up to 100 mM), ammonium acetate (NH_4_Ac), and Tris-HCl were identified as optimal choices for SEC-ICP-MS mobile phases. Nonetheless, the issues with recovery indicate that even in optimal conditions, metal redistribution (not only within samples but also between a sample and a stationary phase) is a common limitation in metalloproteomic speciation studies of labile metal ions. As an additional precaution from these observations, it can be recommended to wash columns with buffers containing an appropriate metal chelator such as EDTA before and after running samples.

Another option for (metallo-)protein separation is 2D gel electrophoresis (2D-GE) [48,49]. Also in this case, native-like conditions are imperative. This means that the dimension that separates according to molecular weight should be a variation of native polyacrylamide gel electrophoresis (PAGE), and the dimension that separates according to charge (typically isoelectric focusing; IEF) should be restricted to a pH range that neither demetallates (competition from protons at low pH) nor denatures the proteins. The drawback of these restrictions is a loss of resolution in both dimensions [6]. An improvement in molecular weight resolution can be achieved using “native SDS-PAGE”, where the SDS (sodium dodecyl sulfate) content is optimised to a minimum level that avoids protein denaturation [50]. Also, 2D-GE has been relatively popular in the past decade, but more recently, LC-based studies have been more frequently reported. However, an innovative approach using 1-dimensional continuous-flow gel electrophoresis in a column format has been developed to identify targets of bismuth drugs in *Helicobacter pylori* [51].

Finally, for the sake of completeness, it may also be mentioned that IMAC (Immobilised Metal Affinity Chromatography) can be used to capture proteins with metal-binding capacity from complex mixtures [52,53]. It is, however, important to understand that this approach gives no information on whether the respective metal ion is bound in vivo.

All approaches mentioned above are, in principle, not only applicable to various biological fluids including serum, plasma, cerebrospinal fluid, milk, and cell extracts, but also applicable to solubilised membrane proteins.

### 2.3. Matching Metals with Proteins

Once an adequate degree of separation has been achieved, the quantitative measurement of the metal ion(s), as well as the identification (and, ideally, also quantitation) of proteins, is required. The latter can be accomplished by standard state-of-the-art MS-based proteomics approaches; typically, this involves bottom-up proteomics, i.e., liquid chromatography tandem MS (LC-MS/MS) analysis of peptides arising from enzymatic digestion. These approaches will not be reviewed here; the reader is referred to recent reviews [54,55,56,57].

For metal quantitation, the overwhelming majority of LC- and CE-based studies involve ICP-MS [11,25,27], with a few examples for Inductively-Coupled Plasma Optical Emission Spectroscopy (ICP-OES) [58] and Graphite Furnace Atomic Absorption Spectrometry (GFAAS) [59]. For metal quantitation for 2D-GE, at least four methods are available: Laser-ablation ICP-MS (LA-ICP-MS) [60], X-ray fluorescence (XRF) [61,62,63], application of a metallochromic dye or fluorescent sensor [64], and autoradiography of proteins labelled with a radioactive metal ion. The latter approach is known as MIRAGE (Metal Isotope native RadioAutography in Gel Electrophoresis) and has yielded a range of new insights into microbial metalloproteomes [65,66]. Nonetheless, it has been noted that only ca. 20% of the predicted zinc metalloproteome was detected using MIRAGE [67], emphasising inherent limitations in detecting low-abundance metalloproteins.

Essential metal ions do not only pose analytical challenges due to the lability of their complexes, but also because their detection is typically less sensitive than that of heavier elements. To a large extent, this is due to the lighter elements being more afflicted by polyatomic and isobaric interferences. Collision and reaction-cell technology has certainly revolutionised the analytical performance of ICP-MS instrumentation, although it is true that instrumental conditions such as the choice of reaction/collision gas require a high degree of expertise. In addition, limits of quantitation can be further improved considerably by employing isotope-dilution analysis [68].

As previously indicated, matching metals to proteins remains challenging, but a few studies have successfully used statistical approaches that track the quantitative co-occurrence of metals and proteins across multiple fractions from two- or multi-dimensional liquid chromatography. Robinson et al. developed a method based on principal component analysis [69]. This approach was used to identify the major copper- and manganese-binding proteins in the periplasm of the cyanobacterium *Synechocystis* sp. PCC6803 [40]. Subsequently, Lancaster et al. developed a computational framework using statistical methodology that enables the prediction of metal–protein associations from quantitative metalloproteomic data (870 proteins, 10 metals (Co, Fe, Mn, Mo, Ni, Pb, W, U, V, and Zn), 2,589 fractions from multi-dimensional liquid chromatography involving multiple column chemistries). This led to the discovery of five novel vanadium-binding, 13 new cobalt-binding, 15 new nickel-binding, and 18 new molybdenum-binding proteins in *Pyrococcus furiosus* [70]—and to the conclusion that “microbial metalloproteomes are largely uncharacterised” [38]. More recently, Mazzotta et al. employed linear regression and singular value decomposition to map the iron [39], zinc, manganese, cobalt, and nickel [71] metalloproteomes of the marine heterotrophic bacterium *Pseudoalteromonas* (BB2-AT2), discussed in more detail in Section 2.5.1.

### 2.4. Alternative Chemoproteomic Approaches

One way to circumvent problems associated with lability harnesses functionalised probes that can form stable covalent bonds between biomolecules and small molecules that interact with them. Such approaches are sometimes referred to as “chemoproteomics” or “chemical proteomics” [72]. Such methodology is gaining traction in the field of drug discovery in general [72] and indeed has already been applied successfully to identify targets for metallodrugs [73,74,75]. This may not even need to involve the interaction of the metal with the protein. Chemoproteomic probes to discover metal–protein interactions are composed of up to three parts: a moiety that binds the metal; a moiety that forms a covalent bond with the protein, often upon photoactivation; and a moiety that allows either immobilisation on a solid phase or attachment to a reporter (e.g., a fluorophore) molecule [76]. Immobilisation allows for the enrichment (“pull-down”) of interacting proteins, whilst fluorescence labelling allows for tracking during separation from complex mixtures. The presence of covalent bonds enables more flexibility and resolution in separation approaches, and the use of standard bottom-up proteomics for protein identification. In addition, it can be possible to image the presence of the probe (and, by inference, the metal) in live cells. Chemoproteomic approaches have been employed to identify targets for Ru-based drugs [73,77] and N-heterocyclic carbene complexes with Au(III), Pt(II), and Pd(II) [76,78]. Target proteins identified for a Rh(I) tricarbonyl complex through chemoproteomics included members of the Fe-S biogenesis machinery [79]. Similar approaches developed by the team of Sun have led to the discovery of large numbers of protein targets for Ni(II) [80], Cu(II) [81], Bi(III) [51], As(III) [82], Fe [83], and Cr(III) [84]. The probes employed are fluorescent metal-specific “tracers”, which consist of a chelating moiety tailored to the metal of interest (with the latter already bound prior to applying the probe to cells), a fluorescent moiety, and an azide group that forms, upon photoactivation, a covalent bond with the proteins that interact with the chelated metal ion.

However, since the metal ion of interest needs to engage several bonds for chelation by the probe, it will have a lower number of bonds available for interactions with proteins; therefore, binding sites requiring more than the available bonds could potentially elude detection. Also, since the metal ion is already part of the probe, this approach is not suitable to determine native metalloproteomes. In these respects, this method has similarities with IMAC (where the immobilised metal is also chelated and supplied externally), but it is superior because the binding event happens in living cells.

### 2.5. Selected Examples for Metalloproteomic Speciation Studies on Labile Metals in Complex Mixtures

Metal speciation studies have been carried out in a range of biological systems. In this section, we provide some examples of these as applied to prokaryotes, plants, and animal biofluids, cells, and tissues. Studies on essential elements as well as Ag(I), Cd(II), Hg(II), and Gd(III) are included. In each case, the respective experimental set-ups and results obtained are highlighted in order to provide insight into experimental conditions that may preserve metal–protein bonds.

#### 2.5.1. Prokaryotic Microbes

Besides the studies on *Synechocystis* and *Pyrococcus furiosus* already mentioned above, this section highlights more recent studies. A review of microbial metalloproteomics is also available [66].

The metalloproteomes for the marine heterotroph *Pseudoalteromonas* (BB2-AT2) for Fe [39], Zn, Ni, Co, and Mn [71] were explored. Cell lysates were prepared anaerobically and subjected first to AEX (single-use GE HiTrap Q HP, 0–1.0 M NaCl gradient), concentrated using ultracentrifugation before SEC (Tosoh Bioscience TSKgel G3000SWxl), with both chromatographies in HPLC format and a 50 mM Tris-HCl (pH 8.8) buffer used throughout. Major Fe-bound proteins identified included, amongst others, bacterioferritins, Fe-superoxide dismutase, catalase, cytochrome c1, and several iron–sulfur cluster proteins [39]. A total of 33 proteins likely to be associated with either Zn, Mn, Co, or Ni were also reported, with the Zn proteome being the overall most dominant, with roles in transcription, translation, proteolysis, and phosphate hydrolysis [71].

The effect of sub-lethal Cu(II) on photosynthetic pigments (light-harvesting complexes and reaction centres) in the purple bacterium *Rhodospirillum rubrum* were studied using analytical metalloproteomics [85]. Reaction centres and light-harvesting complexes (both of which reside in membranes) from bacteria treated with different amounts of Cu(II) were isolated and kept solubilised by a small amount of the detergent n-dodecyl-β-D-maltoside (DDM). Magnesium (the natural cofactor in bacteriochlorophyll) and copper binding was studied using a setup (HPLC-DAD-sfICP-MS; high-performance liquid chromatography-diode array detector-sector-field-ICP-MS [86]) that allowed for correlating metal quantitation with absorbance spectra acquired during an SEC elution. Sufficient resolution was achieved by employing three SEC columns (10 × 300 mm; Superdex Increase or Superose Increase, Cytiva; 150 mM NH_4_HCO_3_, and 0.2 mM DDM) in series. The study demonstrated that the exposure of the bacteria to Cu(II) concentrations as low as 2 mM led to the degradation of photosynthetic reaction centres, with the degradation products harbouring bound Cu.

A 2D separation approach with weak AEX (HiPrep 16/10 DEAE FF, GE Healthcare; 20 mM Tris-HCl (pH 7.4) and 0–1.0 M NaCl gradient) in the first dimension and 1D-GE (25 mM Tris, 192 mM glycine, 0.1% SDS, pH 8.3 as running buffer and 50 mM NH_4_NO_3_ as elution buffer) in the second dimension was developed and used to study metalloproteins in *E. coli* [87]. Two Mg-containing membrane proteins were identified (CorA and MgtA), along with periplasmic copper-containing CueO and zinc-containing ZraP (periplasmic) and DnaJ (cytosolic). Surprisingly, a silver-containing ribosomal protein was also detected, but no complexes with other metals were evident.

**Figure 3:**
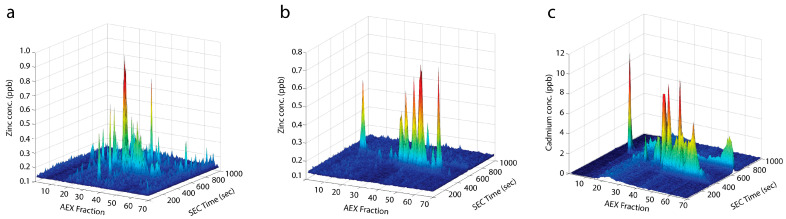
Zinc and cadmium distribution in *S. pneumoniae* proteomes. (**a**) Zn(II)-bound proteins in untreated bacteria. (**b**) Zn(II)-bound proteins in Cd(II)-treated bacteria. (**c**) Cd(II)-bound proteins in Cd^2+^-bound bacteria. Reproduced from reference [88] with permission.

As part of a study on cadmium stress in *Streptococcus pneumoniae* transcriptomics, metabolomics and metalloproteomics were combined. For the latter, cell lysates (20 mM Tris-HCl pH 8.0) were fractionated using 2D LC-ICP-MS, with AEX (Agilent Bio IEX) as first dimension and SEC (Agilent SEC-3) directly hyphenated to ICP-MS as second dimension [88]. Elemental maps were acquired for both Zn(II) and Cd(II), with several hundred peaks observed (Figure 3). Proteins in metal-containing peaks were identified through bottom-up proteomics. A clear loss of zinc-containing proteins was observed in Cd-treated cells. Cd(II) was found associated with many metalloproteins, consistent with the displacement of Mg, Zn, Mn, and Fe.

A recent study developed a workflow for the analysis of large membrane-bound complexes involved in ammonium oxidation and carbon fixation from the bacterium “*Candidatus Kuenenia stuttgartiensis*” strain CSTR1 [89]. This involved the solubilisation of these complexes using mild detergents and separation through 2D gel electrophoresis (first dimension: blue native GE; second dimension: SDS-PAGE). Metals were transferred to a triple-quadrupole ICP-MS instrument either using laser-ablation of dried gels or through a nebuliser system after total digestion of gel slices. The presence of expected metals (Fe, Cu, Zn, Mo) in the high-molecular weight fraction was demonstrated. The desolvating nebuliser approach showed superior performance in detecting Ni but was associated with a more elaborate sample preparation.

Li et al. tested three 1D-PAGE versions for suitability to detect and identify Ag-binding proteins: normal SDS-PAGE, native PAGE, and “native SDS-PAGE” [90]. Normal denaturing SDS-PAGE (involving treatment with β-mercaptoethanol and heating) led to the loss of Ag from proteins; native PAGE suffered from poor resolution, but native SDS-PAGE (0.125 M Tris-HCl pH 6.8, 50% (*v*/*v*) glycerol, 2% (*w*/*v*) SDS, 100 mM TCEP (Tris(2-carboxyethyl)phosphine), 0.02% (*v*/*v*) bromophenol blue) led to clear bands whilst preserving Ag-protein complexes. Cell lysates of *Pseudomonas aeruginosa* and *Staphylococcus aureus* exposed to silver nanoparticles were analysed using this method, leading to the identification of three Ag-bound proteins in either bacterium.

Another investigation of Ag(I) in *E. coli* and *S. aureus* employed SEC-ICP-MS (Agilent AdvanceBio SEC 300 Å, 300 × 7.8 mm, particle size: 2.7 µm; 100 mM HEPES) and identified Ag bound to high-molecular-weight (20–1220 kDa; note that whilst the correct unit of molecular weight is g mol^−1^, it is universally common practice in protein science to refer to “molecular weight” using the unit of “kDa” (which is the unit for molecular mass). To avoid uncommon terminology, we have kept to this terminology.) species of cell lysates, with notable absence of Ag in the low-molecular-weight fraction—thus eliminating Ag complexation with small intracellular thiols such as glutathione (GSH) or bacillithiol [91]. Complementary X-ray absorption spectroscopy (XAS) experiments showed Ag to be bound by S-donor atoms in both bacteria and in lysogeny broth (exclusively bound to an unidentified 30–50 kDa species in broth). Interestingly, the analysis of bacterial cell lysates showed related but not identical ^107^Ag and ^65^Cu elution profiles, consistent with previous works reporting the upregulation of copper transport/homeostatic proteins induced by the presence of Ag.

Using 2D LC (Mono P 5/50 GL FPLC column, 25 mM Tris-HCl and polybuffer 74 or 96 mobile phase) and continuous-flow 1D-GE in the column format (reverse multilayer native resolving gel (13 to 10%) and 4% stacking gel) coupled to ICP-MS (LC-GE-ICP-MS), bottom-up proteomics, and bioinformatics, 34 Ag-binding proteins were identified in *E. coli* cell lysates. Ag(I) was found to perturb several enzymes involved in glycolysis and the tricarboxylic acid (TCA) cycle, causing systemic damages and ultimately leading to cell death [92].

#### 2.5.2. Plants

Various analytical approaches have been used to examine metal speciation in plants, seeds, and nuts, largely focusing on small-molecule complexes rather than metalloproteins. For such studies, several recommendations have been made for sample collection [93] that are also applicable for metalloproteomic studies. These include collecting specimens in acid-washed plastic containers using tweezers made from a suitable material (e.g., nylon, ceramic, or Teflon). Samples close to the ground (e.g., stems, leaves, or bark) should be avoided, or if this is not possible (e.g., in the case of roots, tubers), they should be washed to limit soil contamination. Some considerations on sample preparation for plant metallomics are given in [94].

Speciation studies related to metalloproteomics in plants began with developing a SEC-ICP-MS approach to quantify Cd(II) complexes with phytochelatins (PCs) [95]. The latter are non-ribosomally synthesised cysteine-rich peptides. The separation conditions, which utilised a 30 mM Tris-based buffer (pH 8.5) for the mobile phase, were optimised using standards prepared by the incubation of Cd(II) with purified (GluCys)_2_Gly (PC_2_), (GluCys)_3_Gly (PC_3_), and (GluCys)_4_Gly (PC_4_) ligands. Cd complexes with PC_2_, PC_3_, and PC_4_ were detectable in all samples investigated, each eluting at the times corresponding to standards. The validation of the method was achieved by comparing the results obtained using SEC-ICP-MS with those obtained using reversed-phase HPLC with post-column derivatisation of phytochelatins.

Several studies employing 1D SEC-ICP-MS approaches to support studying metal toxicity in plants are available from the Küpper team, including Cu [96] and Cd toxicity in the aquatic macrophyte *Ceratophyllum demersum* [97] and Cd toxicity in soybean [98,99]. Chromatographic resolution was boosted by using two or three SEC columns in series. NH_4_HCO_3_ buffer (150 mM, pH 7.8) was suitable for either soluble or, with the addition of detergent (e.g., 0.2 mM DDM), membrane proteins. Proteins in selected fractions were identified using standard MS-based bottom-up proteomics. In *Ceratophyllum*, primary targets of both Cu and Cd toxicity were components of the photosynthetic machinery. This indicated that both metals were capable of displacing Mg in chlorophyll, leading to the degradation of light-harvesting complexes, especially those for photosystem II. The latter were also impaired in soybean under chronic Cd exposure [99]. In soybean roots, Cd(II) was found to be bound to aquaporins and peroxidases [98].

#### 2.5.3. Blood Plasma/Serum

Arguably, the field in which metalloproteomics has been most prolific is biomedical science, with blood serum or plasma being particularly popular in the study of both essential and xenobiotic metals (also see Section 3). The circulatory system is responsible for the distribution of essential metal ions throughout the body (once these have arrived in the portal vein after absorption in the gut). In blood plasma, most metal ions are, at least to some degree, bound to proteins that facilitate their transport. Abnormal concentrations of individual metals are often used to indicate metal status (i.e., deficiency, sufficiency, or toxicity), while altered speciation may indicate defects in metal handling and homeostasis and be associated with disease. In addition, plasma or serum are amenable to metalloproteomics approaches with minimal sample preparation (and, hence, perturbation). For these reasons, there is a large body of work on plasma or serum metalloproteomics.

Several studies by the Gailer team employed 1D SEC in FPLC format of plasma and serum. Building on previous metalloproteomic work [46,100], an approach that utilises the lability of metal–protein complexes to further understand plasma metal speciation was developed [58,101]. The analytical method involves SEC (Superdex^TM^ 200 Increase 10/300 GL, Cytiva; 150 mM PBS pH 7.4) hyphenated to ICP-OES. In these experiments, human plasma was spiked with either caeruloplasmin, holo-transferrin, or α2-macroglobulin. This aided in the identification of the endogenous fractions that contained these proteins and, in each case, revealed that the binding of the respective metal to these proteins was favoured over other plasma proteins. In addition, the analysis of fractions obtained from SEC revealed that spiking altered the distribution of metals in fractions, such that Cu, Fe, and Zn were sequestered from other endogenous protein fractions to those containing the added caeruloplasmin, transferrin, or α2-macroglobulin, respectively. Recently, the same lab explored the merits of SEC-GFAAS for plasma metalloprotein analysis [59]. The methodology benefits from smaller sample requirements and higher sensitivity than the previously employed SEC-ICP-OES approach and may be applicable to clinical samples.

**Figure 4:**
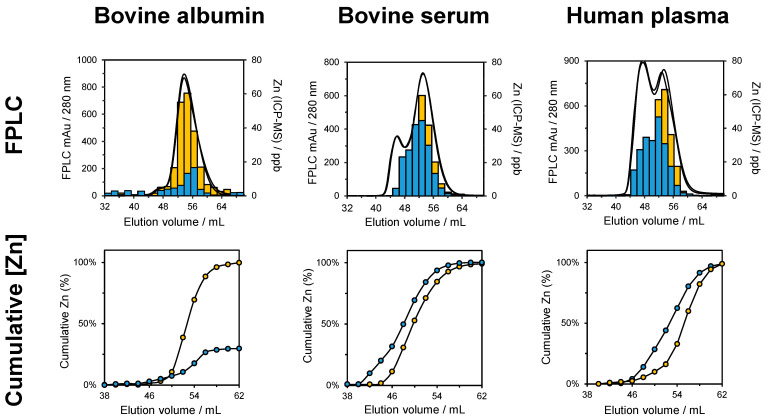
Results obtained from a 1D metalloproteomic study evidencing Zn(II) redistribution as a consequence of fatty-acid loading of albumin [102]. The black lines refer to absorbance at 280 nm; the yellow bars and points refer to Zn levels in the absence of myristate; the blue bars and points refer to Zn levels in the presence of 3 mM myristate, expected to be fully bound to albumin (ca. 600 μM). In the absence of other proteins, Zn(II) transfers from BSA to the column material (**left panel**). When 3 mM myristate is added to serum (**middle panel**) or plasma (**right panel**), Zn(II) displaced from albumin transfers to other proteins with higher molecular weight (lower elution volume).

We recently examined the effect of fatty acid (myristate) on the speciation of Zn in serum and plasma [102]. Under normal conditions, ~75% of Zn(II) in plasma is bound to serum albumin, which aids its transport in the body [103]. Albumin also transports fatty acids through binding at up to seven specific binding sites [104]. It is known that elevated levels of plasma fatty acids, as observed in some disease states, can reduce the ability of albumin to bind Zn(II) through an allosteric mechanism. Using offline FPLC-SEC (Superdex G75, Cytiva; 50 mM NH_4_Ac pH 7.8) and ICP-MS, we examined the effects of adding 3 mM myristate on Zn (20 μM) speciation in a bovine serum albumin (BSA) solution (300 μM), 50% foetal calf serum (FCS), and 50% human plasma (Figure 4) [102].

The ICP-MS analysis of SEC fractions from the BSA solution determined that the addition of myristate significantly decreased the amount of albumin-bound Zn by 69.9% (from 20 to 6.02 μM) relative to the FFA-free control. The SEC fractionation of FCS revealed that myristate invoked a re-distribution of Zn, predominantly from albumin-containing fractions to higher-molecular-weight species. This finding was replicated in human plasma following myristate addition. Using immunodetection, candidate acceptor proteins for Zn(II) lost from albumin were found to include histidine-rich glycoprotein (HRG), a multifunctional protein associated with the regulation of blood coagulation. In a separate workflow, plasma proteins with zinc-binding ability were captured using Zn-IMAC and identified using peptide mass fingerprinting. Captured proteins included members of the complement system, which is involved in the innate immune response. Collectively, the findings suggested that fatty acid-mediated changes in plasma metal speciation could contribute to the progression of certain pathological conditions associated with abnormal circulatory Zn(II) handling (e.g., diabetes [105], thrombosis [106], and neurodegenerative diseases [107]).

SEC-ICP-MS was also employed to study copper speciation and the dynamics between plasma proteins previously reported to bind Cu(II), including HSA, caeruloplasmin, and α2-macroglobulin [108]. The SEC column used was based on desalting resin (Sephadex G25, GE Healthcare), with the aim to separate high- from low-molecular weight complexes. Proteins, Cu(II), and small-molecule ligands (histidine, glutamate, glycine, NTA, and EDTA) were mixed and eluted with 50 mM HEPES, 50 mM NaCl, pH 7.4, and Cu in peak fractions was quantified using ICP-MS. This methodology allowed for studying the thermodynamics and kinetics of Cu(II) binding. HSA was found to exhibit low pico- to femtomolar affinity, depending on competing ligands. Cu(II) bound to caeruloplasmin was inert, so equilibrium constants could not be established. Contrary to previous reports, no significant Cu(II) binding to α2-macroglobulin was found.

Wilson’s Disease (WD) is a genetic disorder that is caused by defects in the transmembrane copper-transporting ATPase protein encoded by the *ATP7B* gene. Predominantly expressed in hepatocytes, this protein is involved in both the excretion of surplus copper as well as pumping copper into the Golgi apparatus for the metallation of caeruloplasmin. The disease is characterised by copper accumulation in the liver, brain, eyes, and other organs [109]. In plasma, WD manifests as a lack of copper in caeruloplasmin (usually the largest pool of non-exchangeable copper in plasma) and increased levels of exchangeable copper, predominantly bound to HSA. Several methods have been developed recently to harness altered copper speciation as a diagnostic tool for the detection of WD. A rapid LC-ICP-MS method based on a type of chelating resin (Elemental Scientific CF-Cu-02) was developed to separate and quantify protein-bound and extractable copper in the serum of a rat model for WD [110]. Plasma was diluted 10-fold with de-ionised water before loading onto the chelating column. Notably, the LC system employed in this study (Elemental Scientific prep*FAST*IC) utilises metal-free syringe pumps to minimise contamination. Column-bound Cu was then eluted first with 100 mM NH_4_Ac, pH 7.4, and then with 10% nitric acid and quantified using ICP-MS. WD rats had a much higher percentage of extractable serum copper (34–38%) than healthy controls (ca. 6%), irrespective of whether diseased or not. The authors suggest that the method may offer a simple and fast clinical test to assess WD patients.

Since both albumin and caeruloplasmin show altered metallation in WD, their copper loading may also be a suitable biomarker for this disease. Accordingly, two separate teams recently studied serum copper speciation in WD patients and healthy controls in further detail [111,112]. Both labs used anion-exchange chromatography coupled to ICP-MS. Solovyev et al.’s approach employed strong AEX (Q-STAT; Tosoh Bioscience), eluted with an NH_4_Ac gradient (with buffering to pH 7.4 provided by 50 mM Tris-HCl) and a triple-quad ICP-MS instrument (SAX-ICP-MS-MS) to quantify Cu-CP and Cu-HSA. Protein concentration was measured through ^16^O^32^S^+^ and ^16^O^34^S^+^ (enabled by the triple-quad mode with O_2_ in the collision/reaction cell) simultaneously with ^63^Cu^+^ and ^65^Cu^+^. Busto et al. also used strong AEX (Cytiva MonoQ™; 50 mM Tris-HCl, 0–250 mM NH_4_Ac gradient, pH 7.4) coupled to ICP-MS to quantify Cu-HSA, Cu-caeruloplasmin, and exchangeable copper (extractable by EDTA). Copper quantitation in protein fractions was validated using a double species-specific HPLC-ICP-IDMS (isotope dilution mass spectrometry) method to quantify Cu-albumin.

Michalke and collaborators developed a large body of work using LC-ICP-MS methodology to study metal speciation in serum (as well as in other specimens such as cerebrospinal fluid (CSF) and brain extracts (see Section 2.5.5), mainly in the context of neurodegenerative disease [113,114]. The two major fractionation techniques used were SEC and AEX, often combining several complementary separation columns. Several studies have used two serially connected SEC columns, one for the separation of high-molecular weight (HMW) species (e.g., Thermo Fisher Scientific Biobasic 300 mesh, separation range 700–5 kDa [115,116], or Tosoh Toyopearl TSK HW 55S [117,118]), and one for the separation of low-molecular weight (LMW) species (e.g., Tosoh Toyopearl HW40S, separation range 100–2000 Da). Column casings based on PEEK (polyetheretherketone) were preferred, having the advantage of being metal-free. Buffers were mainly based on NH_4_Ac, sometimes also including small amounts of Tris and methanol, and, typically, samples were introduced in undiluted form to minimise the perturbation of equilibria. Depending on the metal under study, serum and other samples typically yielded between five and nine molecular weight fractions. Major metal-binding proteins in such fractions were assigned through a comparison with authentic standards such as α2-macroglobulin, transferrin, caeruloplasmin, and albumin. For example, manganese speciation in the sera of Mn-exposed rats were studied using SEC-ICP-MS as a model for Parkinson’s disease [117]. Using a buffer composed of 90% 50 mM NH_4_Ac, pH 5.8, and 10% 10 mM Tris, 50 mM NH_4_Ac, 5% (*v*/*v*) MeOH, pH 8, six fractions with different molecular weight ranges were observed and assigned to α2-macroglobulin, γ-globulin, transferrin, citrate, amino acids, and inorganic Mn(II), respectively. In both subchronic (feeding experiment) and acute (intravenous injection experiment) manganese poisoning, the fraction containing amino acids was most significantly affected. Together with an analysis of the brain metabolome, this led to the conclusion that this fraction plays a major role in Mn trafficking within and from serum, and that understanding Mn speciation is critical to understanding the effects of Mn-induced neurotoxicity.

Using a similar approach, Ajsuvakova et al. assessed the speciation of Mn, Fe, Zn, and Cu in the sera of Parkinson’s disease patients [118]. The mobile phase was 50 mM NH_4_Ac at pH 5.8. Since this pH is rather lower than physiological, it may be expected that some native-metal complexes could dissociate under this condition, at least those of the metals lower in the Irving–Williams series (Mn(II), Fe(II)). An increase in LWM copper species was observed in Parkinson’s disease patients.

Speciation analysis using SEC-ICP-MS for Fe, Mn, and Zn in paired serum and CSF from patients with unspecific neurological complaints was also performed using an approach employing two SEC columns [116]. Here, the SEC buffer consisted of 10 mM Tris-HAc and 250 mM NH_4_Ac (pH 7.4). This yielded nine fractions for each metal. In general, there was a trend towards higher proportions of LMW species in CSF compared to serum. Unexpectedly, the major zinc fraction from serum was in the 400–600 kDa range, even though the majority of Zn(II) (75–90% [119]) would be expected to elute with albumin. In the paired CFS samples, ca. 40% of Zn(II) eluted with the albumin fraction.

Using a single Agilent Bio SEC-5 column and a complex elution buffer containing 50 mM NH_4_Ac, 11 mM Tris-HAc, and ca. 2% methanol, Mn, Fe, Zn, and Cu speciation in Fe-deficient rats was examined [120]. Fe deficiency was induced with a desferrioxamine injection. This study suggested that the increase in Mn and the decrease in Fe in the fraction assigned to ferritin indicated incorporation of Mn in ferritin, whilst other studies (e.g., [116,117]) suggested that the Mn species in this molecular weight range correspond to α2-macroglobulin. This discrepancy could perhaps be resolved with a second separation dimension.

Cu and Zn speciation in the serum of dairy cows was explored using SEC-ICP-MS in HPLC format (Agilent Bio-SEC5; 5–1250 kDa) [121]. The mobile phase (pH 7.4) contained NH_4_Ac, Tris-HCl, and 5% methanol. Five Cu and four Zn peaks were observed. Based on comparisons with commercial standards, two peaks were assigned to α2-macroglobulin (tetrameric and dimeric, containing both Zn and Cu), one to caeruloplasmin (Cu only), one to albumin (containing both Zn and Cu), and the last one to LMW species (<1% Cu; 3% Zn). A similar approach was also applied to Mn speciation in bovine serum, finding six peaks presumed to correspond to various forms of α2-macroglobulin, transferrin and/or albumin, and LMW species, with the latter making up about half of the Mn speciation [122].

Garcia-Fernandez et al. studied the fate of iron-sucrose nano-particles that are used to treat iron deficiency in patients with chronic kidney disease [123]. The nano-particles were incubated in serum or blood, and speciation was determined by strong AEX (Cytiva Mono Q 5/50 GL) coupled to ICP-MS. Most solubilised Fe was bound to transferrin, but when the latter’s binding capacity was exceeded, other proteins, possibly albumin, were also associated with Fe. AEX did not only allow distinction between apo- and holo-transferrin, but also between differently sialylated proteoforms of transferrin (holo-forms only). The mobile phases were based on 20 or 50 mM Tris-HAc (pH 7.4), and various NH_4_Ac gradients were used for elution.

Cobalt speciation in serum has also been studied using SEC-ICP-MS [124]. A GE Healthcare Superdex Peptide 10/300 GL column was used for separating high- and low-molecular weight complexes. In undiluted serum, ca. 90% of Co(II) was bound to the HMW fraction, presumed to be dominated by albumin. This study is relevant to the speciation of elevated Co(II) levels as a result of certain hip prostheses as well as to the clinical “albumin-cobalt-binding” assay, which detects the biomarker “ischemia-modified albumin”.

As an alternative to more common separation techniques, asymmetric flow field-flow fractionation (AF^4^) was trialled for the separation of cobalt and chromium complexes from serum and synovial fluid (hip aspirate) of patients with metal-on-metal hip replacements [125]. Whilst AF^4^ separation was inferior to strong AEX, the association of Cr(III) with transferrin and albumin, and of Co(II) with albumin and one or two unknown proteins, was detected and confirmed through AEX. An earlier study using AEX had come to similar conclusions [126].

The identification of uranyl (UO_2_^2+^)-binding proteins is key to the development of efficient detoxification approaches following exposure. Therefore, the binding of uranyl to serum proteins was examined by Huynh et al. [127]. In this study, uranyl was added to serum to a final concentration of 1 μM, and the proteins were fractionated through capillary electrophoresis (CE) prior to ICP-MS analysis. Phosphate buffer was deemed unsuitable due to the formation of phosphate complexes with low solubility. Various combinations of Tris-HCl and NaCl at different pH values were explored as a background electrolyte, with 10 mM Tris and 15 mM NaCl at pH 7.4 considered optimal. Two main uranyl-containing fractions were identified. The first fraction was attributed to the binding to fetuin-A, and, to a lesser extent, to albumin. The second fraction corresponded to non-protein bound uranyl. CE-ICP-MS was also used to investigate the properties of fetuin-A–uranyl complexes enabling the determination of an association constant (K_d_ ~30 nM) and suggested that different species can form dependent upon the carbonate concentration [127].

#### 2.5.4. Other Biofluids

As an extracellular biofluid with certain similarities to serum, CSF has also been subjected to metalloproteomic studies in the context of neurochemistry and metal-related neurodegenerative diseases (e.g., Alzheimer’s, Parkinson’s, and amyotrophic lateral sclerosis) [128,129]. Besides the studies already mentioned in Section 2.5.3 [115,116,117,118], a case–control study focused on metal speciation in CSF of patients with Parkinson’s Disease [130]. Whilst speciation patterns as such did not discriminate between cases and controls, the ratio of total Fe to the Cu amino acid fraction was significantly increased in cases.

Another biological fluid recently studied using metalloproteomic techniques is milk. Zinc speciation in the whey fraction of human breast milk (after full-term and pre-term births) and formula milk was explored via SEC-ICP-MS using a Cytiva Superdex 200 10/300 GL column with 100 mM Tris-HCl (pH 7.0) as buffer [131]. The column was calibrated with ferritin (450 kDa), immunoglobulin G (160 kDa), albumin (67 kDa), ovalbumin (43 kDa), β-lactoglobulin (36.6 kDa, dimeric form), and α-lactalbumin (14.2 kDa). The elution profile showed at least five peaks. A large percentage of Zn(II) was eluted at very high molecular weight, suggested to correspond to immunoglobulins. The proportion of Zn(II) in this fraction was higher for milk after pre-term births. Another large fraction was LMW complexes (<10 kDa; suggested to include citrates), which were higher in full-term samples (48% compared to 32% in pre-term samples), and even higher in formula milk (up to 60%). A similar study looked at iron, copper, zinc, and iodine speciation, again in whey from human breast milk [132]. The SEC separation involved a Cytiva Superdex™ 200 Increase 10/300 GL column and 10 mM Tris-HCl buffer (pH 7.0). Also here, a significant proportion of the metals eluted in several fractions containing HMW proteins, such as immunoglobulins, albumin, and lactoferrin. In contrast, iodine was present as iodide only. A similar approach attempted to study Mn, Co, Cu, and Se speciation [133]. SEC was performed using a TSK gel G3000SW column and 50 mM Tris-HCl buffer (pH 6.8). In this case, only two fractions were resolved, and even though the samples were also subjected to native PAGE and MALDI-TOF/TOF analysis, there was no attempt to correlate specific proteins with metals.

The inner ear of fish contain otholiths (“hearing stones”), and their growth medium—called endolymph—has been studied using SEC-ICP-MS [134]. SEC involved 200 mM NH_4_NO_3_ (pH 7.7–7.8) and an Agilent Bio SEC-3 column. Elution profiles for 23 metals were acquired. Alkali metals eluted exclusively in the salt fraction; earth alkali metals, except for Mg, were found in both protein-bound form and salt fraction; d-block metals (Fe, Co, Ni, Cu, Zn) eluted in one to four protein-bound peaks with none of these metals detected in the salt fraction. Furthermore, V, Hg, As, Sn, and Pb were also found bound to proteins. In each case, metal-associated proteins await identification.

#### 2.5.5. Animal Cells and Tissues

Metal speciation is also investigated in cell models and tissues, often as a means to understand disease mechanisms. Also here, the field of neurochemistry is particularly prolific [12,114,135].

Proteins bound to Cu, Fe, and Zn in primary neuron and astrocyte cell lysates were profiled using SEC-ICP-MS (Agilent Bio-SEC 3; 200 mM NH_4_NO_3_ (pH 7.7–7.8)) [42]. Cell pellets were resuspended in Tris-buffered saline containing EDTA-free protease inhibitors to minimise protein degradation. The neurons and astrocyte extracts exhibited distinct metalloprotein profiles, featuring both ubiquitous and unique metalloprotein species. Most Fe was found bound to ferritin in both cell types, with astrocytes containing as much as 10 times more ferritin-bound Fe than neurons. The Zn profile in both cell types was similar, except for a prominent peak at 4.1 kDa that was only observed in astrocytes and most likely corresponds to metallothioneins. Another peak with co-elution of Cu and Zn was Cu,Zn-superoxide dismutase, present in both cell types. In contrast, caeruloplasmin was observed almost exclusively in neurons.

Total Fe, Cu, and Zn, and their distribution (by LA-ICP-MS) and speciation (by SEC-ICP-MS; Bio SEC-3, 200 mM NH_4_NO_3_, pH 7.5) were studied in a mouse model of tauopathy (diseases associated with tau protein neurofibrillary tangles such as Alzheimer’s disease) [136]. Some metal-containing peaks were assigned to proteins based on previous work using purified standards (thyroglobulin, ferritin, ceruloplasmin, Cu/Zn SOD, and vitamin B12) [137]. All three metals were increased in the disease model mice compared to wild-type controls, and their speciation was also altered, with a trend towards less protein-bound metal. A study by the same team of labs using the same approach found that mice that lack the zinc transporter ZnT-3 had altered speciation for not only Zn, but also Fe and Cu [138].

The study regarding paired human serum/CFS samples described in Section 2.5.3 also included analysis of brain extracts from Mn-treated rats [116]. The already described two-column approach showed that Fe-ferritin (400–600 kDa fraction) was decreased in Mn-exposed rats. The same fraction also experienced an increase in Mn, which was attributed to α2-macroglobulin. In contrast, no significant changes in the distribution of Zn-containing protein fractions between Mn-exposed and non-exposed animals was apparent. The authors suggest that these findings point to a substantial interplay between Fe and Mn homeostasis.

An intriguing study looked at Cu isotopic ratios of Cu,Zn-SOD1 and metallothioneins (MTs) extracted from post-mortem human brains [139]. The proteins were separated using SEC (Cytiva Superdex G75 10/300, 200 mM NH_4_NO_3_, pH 7.7), digested using microwave treatment, and Cu was purified from digests via AEX. Isotopic abundances were measured using a multi-collector ICP-MS. Relative to the average ^65^Cu/^63^Cu ratio, that of MT was lower, whilst that of SOD was higher. The authors concluded that nitrogen ligands (as in SOD) promote the accumulation of heavier isotopes, whilst sulfur ligands (as in MTs) promote the binding of lighter isotopes.

The disruption of iron homeostasis has been suggested to be a hallmark of cancer progression, prompting a study into iron speciation in two breast cancer cell lines [140]. Total Fe quantitation and speciation analysis of the >30 kDa fraction of cytosols using SEC-ICP-MS (conditions were not available) revealed that the more aggressive cell type had lower total Fe as well as a lower proportion of ferritin-bound Fe, even though total ferritin was higher. The latter high-accuracy quantitation was achieved by using ICP-MS (with isotope dilution analysis; IDA) readings for Ru after labelling ferritin with a Ru(bipy)_3_-tagged antibody.

The nematode *Caenorhabditis elegans* is frequently used as a model organism in developmental biology. One SEC-ICP-MS-based study explored the changing metalloproteomic profiles for Fe, Cu, and Zn in eggs, four larval stages, and young adult worms [141]. Soluble metalloproteins were extracted using Tris-buffered saline containing an EDTA-free protease inhibitor cocktail. SEC involved an Agilent Bio SEC-5 column and 200 mM NH_4_NO_3_ (pH 7.7–7.8). For Fe, ferritin (by comparison with an authentic standard) was shown to vary with developmental stage, being lowest in eggs and highest in young adults. Elution profiles for Cu and Zn also changed, but the associated proteins could not be identified conclusively.

Besides studies on essential metal ion complexes, there is also considerable interest in understanding environmental toxicity in animals and humans. In particular, mercury (Hg) bioaccumulation in aquatic species poses a significant toxicological risk in human populations with high seafood consumption. Whole-tissue concentrations of Hg ranging between 4.4 and 317.4 ng⋅g^−1^ have been determined in tuna and salmon tissues. SEC-ICP-MS (Sepax Technologies Nanofilm SEC-150 column (4.6 × 300 mm, 5 μm); 50 mM Tris and 50 mM NH_4_NO_3_ pH 7.5) was used to determine Hg speciation. Around 30.2–37.6% of the total Hg was found to be bound to β-actin [142]. In another study, the muscle and liver tissues of *Serrasalmus rhombeus* (black piranha) were analysed to identify proteins as bio-markers for their Hg exposure. The proteome was separated using a standard 2D GE approach, and Hg was quantified using GFAAS. From Hg-containing spots, nine proteins were identified by ESI-MS/MS [143]. These proteins included haemoglobin and several Mg- or Ca-binding proteins. The latter study may serve as an illustration that at least some complexes with Hg(II) can tolerate the wide pH range used in isoelectric focusing as well as protein denaturation associated with SDS-PAGE that are applied in standard 2D GE.

Target proteins for uranyl in zebrafish gills were explored using off-gel IEF (pH 4–7 [144]) followed by SEC-ICP-MS using a sector-field MS. Gill homogenates were prepared in 10 mM NH_4_Ac (pH 7.4), subjected to non-denaturing IEF, and then each of the 10 IEF fractions was separated using SEC in HPLC format (Cytiva Superdex 200 10/300 GL, 600–10 kDa, 300 × 10; 100 mM NH_4_Ac). Proteins in U-containing IEF fractions were identified using both bottom-up and top-down proteomics, giving some potential leads for U-binding proteins. A low recovery of U from the 2D separation approach was noticed, and it was suggested that the conditions chosen may only maintain the strongest uranyl-protein complexes [145].

Plutonium-binding proteins from rat adrenal gland cells were captured by IMAC using NTA-immobilised Pu(IV) [146]. Proteins recovered from IMAC eluates were further separated using standard 2D-GE and subjected to bottom-up proteomic identification. Seven new Pu-interacting proteins were identified, most of which were native metalloproteins and some of which were anti-apoptotic—suggesting new lines of investigation regarding Pu-induced cancer.

#### 2.5.6. MRI Contrast Agents and Related Lanthanide Compounds

Although MRI contrast agents are designed to be inert with respect to the dissociation of the metal ion from the chelating ligand, lanthanide complexes per se are labile; thus, studies aimed at identifying the proteins they interact with require careful experimental design [147,148,149,150,151]. Several studies have been directed towards understanding gadolinium speciation in the context of Gd(III) retention in the brain, as reviewed in [152]. This is a particular problem for Gd(III) complexes with linear polydentate ligands, which undergo dissociation more easily than complexes with macrocyclic ligands. Such studies typically entail the determination of total amounts of Gd in various brain regions, but, more recently, speciation analysis has been deployed as well. Analyses of soluble fractions from various brain extracts of animals exposed to such complexes have repeatedly shown the presence of Gd complexes with (usually unidentified) macromolecules. This is exemplified by a study by Frenzel et al., who found Gd bound to high-molecular weight proteins (250–300 kDa) in homogenates of cerebellum from rats treated with clinically used linear agents (gadodiamide, gadopentetate, dimeglumine) 24 days after the last administration, while no such effect was found with macrocyclic contrast agents (gadobutrol and gadoterate meglumine) [153]. The SEC-ICP-MS approach involved a Superdex 75 HR HPLC column and a buffer of 10 mM Tris and 40 mM ammonium formate at pH 7.4. Another study found that an entire year after the last injection of a linear contrast agent (gadodiamide), 75% of the Gd measured in the cerebellum was bound to soluble proteins (>66.5 kDa), and that this proportion increased over time [154]. Again, this was not the case for the macrocyclic gadoterate.

Three recent related studies by a single team explored various aspects of Gd retention [150,155,156]. After a single injection of linear agents, Gd was found bound to soluble macromolecules (>80 kDa), even after 5 months [155]. A subsequent study in which brain tissue was solubilised with urea found Gd in two HMW fractions, >660 kDa and 440 kDa, with the latter suggested to be ferritin, based on comparison with a ferritin standard. The study also demonstrated that Gd(III)-ferritin complexes can form and be detected by the SEC-ICP-MS approach used, which involved a Superdex 200 column and 100 mM NH_4_Ac (pH 7.4) [156]. The same species were also found in the water-soluble fraction of deep cerebellar nuclei of rats that had received four daily doses of contrast agent over a period of 5 weeks, again exclusively after the administration of the linear gadodiamide [150].

Another biomolecule suggested to interact with retained Gd is tubulin, as identified through a combination of SEC-ICP-MS, SDS-PAGE, and LC-Triple TOF MS [157]. The cells used in this study were mouse fibroblasts (NIH-3T3). The SEC separation employed a Zenix SEC-300 (MW range 0.5–150 kDa; Sepax Technologies) column, with a mobile phase of 50 mM NH_4_Ac (pH 7.0). The majority of Gd seemed to have eluted in the void volume, with further separation of this fraction through SDS-PAGE revealing one band with high Gd content and the respective protein identified as tubulin.

Lanthanides can also be used for labelling proteins, for example antibodies. Ultra-HPLC-SEC-ICP-MS (Acquity UPLC Protein BEH (Ethylene-bridged hybrid) SEC column; 100 mM NH_4_Ac, pH 6.8) with post-column IDA (for lanthanides Eu, Dy, or Gd and sulfur) has been developed [158]. This SEC-IDA-ICP-MS method can be used to study labelling efficiency, antibody integrity, and to quantify immunoassays after the antibody has bound target proteins.

## 3. Inert Complexes

**Figure 5:**
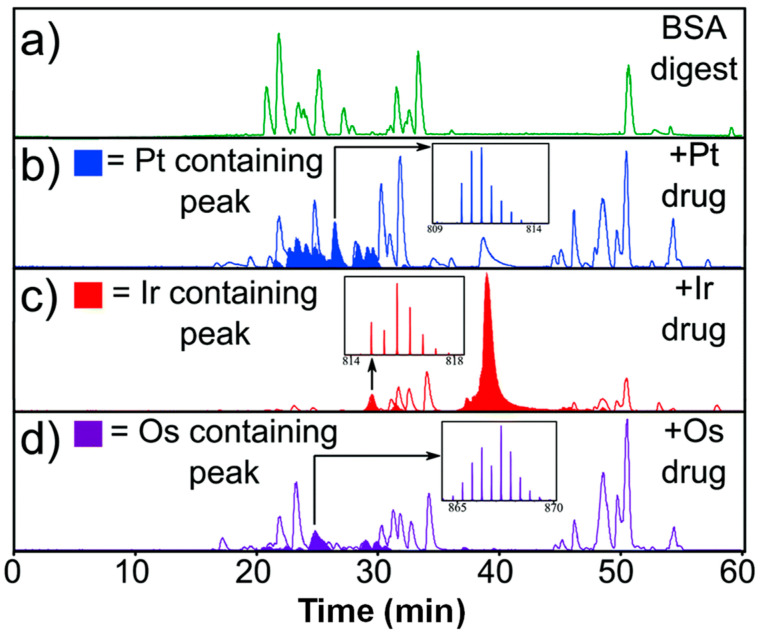
Observation of metallated peptides in digests of BSA (**a**) before and after reaction with metallodrugs (**b**) *trans*,*trans*,*trans*-[Pt(N_3_)_2_(OH)_2_(py)_2_], (**c**) [(η^5^-Cp*)Ir(2-(R’-phenyl)-R-pyridine)Cl], and (**d**) [(η^6^-bip)Os(en)Cl]^+^ (where bip = biphenyl, and en = ethylenediamine). Reproduced from reference [162] with permission.

As previously indicated (Figure 1), some 4d and 5d transition metal ions form inert complexes, as indicated by very slow water exchange rates (<10^−3^ s^−1^). The most active area where metalloproteomics is harnessed for studying the interactions of these metal ions with proteins concerns the mode of action and fate of metallodrugs [75,159,160,161]. It may be noted that although Pt(II), Ru(II), Ru (III), Os(II), and Os(III) form strong bonds and have relatively slow exchange rates (Figure 1), metallodrugs based on these metal ions are designed to have a degree of reactivity, often by including a leaving group such as chloride that readily exchanges with other ligands including proteins and DNA. In contrast to the mode in which essential metal ions are bound, the resulting complexes often involve just one bond between metal and biomolecule. Nonetheless, as documented below, such complexes are often, but not always, quite stable and inert, and they may even permit their detection through bottom-up proteomics approaches (which involve the denaturation and digestion of the protein(s) under study). These are facilitated by the unique isotopic patterns of metal ions such as Pt, Ru, Os, and Ir, which allow for the unambiguous detection of metallated peptides (Figure 5) [162]. The Smart Numerical Annotation procedure (SNAP) allows for an automatic assignment of these.

These xenobiotic heavy metals have several further properties that facilitate metalloproteomic analysis. Firstly, their detection limits in ICP-MS tend to be higher than those for the lighter elements, owing to the absence of polyatomic and isobaric interferences. Secondly, contamination with ambient levels of the same element is much rarer, in contrast to metals such as zinc and iron which are ubiquitous in a standard lab.

The application of metalloproteomic techniques to metallodrug development has provided a wealth of mechanistic insight, including the identification of protein targets [75] and/or deactivation pathways, and facilitates the translation of metallodrugs to clinical investigation [161]. As our review focuses on the importance of kinetic (and thermodynamic) properties of metal–protein complexes and their impact on analysis, we limited the reviewed material to approaches that involve the detection of these complexes.

### 3.1. Platinum Complexes

Up to half of all chemotherapy treatments include a platinum drug, with cisplatin, carboplatin, and oxaliplatin being notable examples. All of these drugs are administered intravenously; therefore, both extra- and intracellular speciation are of interest. The following studies illustrate that Pt(II)-protein adducts can, in principle, be analysed successfully under much harsher conditions than those with labile metal ions. MS-based investigations into interactions between platinum drugs and plasma proteins over the past three decades have been summarised recently [163]; therefore, only a few studies are highlighted here.

The applicability of bottom-up techniques to identify Pt binding sites on proteins has been established in several studies. Will et al. used multidimensional liquid chromatography and ESI tandem mass spectrometry (MudPIT) on cisplatin-blood serum incubations [164]. Reaction mixtures were digested with trypsin, and the resulting peptides were separated by strong cation exchange and reverse-phase HPLC. Fractions were subjected to tandem ESI-MS with the aim to detect platinated peptides. Albumin, transferrin, α2- macroglobulin, α-1-antitrypsin, and apolipoproteins A1 and A2 were found as binding partners. The technique also allowed for the pinpointing of binding sites. For example, those for albumin included Cys34, two methionine residues, and several sites formed by O-donors.

Following on from previous work dedicated to establishing conditions for gel-based workflows that preserved Pt-protein bonds [165,166], Pt-protein adducts of several Pt(II)-drugs with selected protein standards and human serum were identified via a shotgun proteomic approach [167]. This involved the tryptic digestion of proteins, fractionation of the digests using off-gel-IEF (pH 3.5–9.5), and detection of platinated peptides using nLC-ESI-LTQ Orbitrap-MS/MS. The application of filter-aided sample preparation (FASP) even permitted the use of DTT (dithiothreitol) without a significant loss of Pt-peptide bonds. A total of 18 platinated peptides, all derived from albumin, were found. This approach works well for high-abundance proteins, with the bonus of enabling the identification of binding sites on the proteins but is unlikely to find adducts with less abundant proteins.

SEC-ICP-OES was employed to investigate the interaction of plasma proteins with novel Pt anticancer complexes of the general formula [(Pt(terpyridine))_2_μ-(X)]^n+^, where X is a linker group [168]. Complexes were incubated (5 min or 60 min) in rabbit plasma prior to SEC-ICP-OES (Superdex 200 10/300 GLSEC column with 150 mM pH 7.4 PBS mobile phase). The identification of specific plasma protein adducts was limited by the SEC resolution; however, the co-elution of Pt with Fe and Zn indicated that the Pt-terpyridine complexes likely formed adducts with transferrin and albumin.

Galvez et al. evaluated four different approaches (SEC-ICP-MS, Ultra-HPLC SEC-ICP-MS, TFC(turbulent-flow-chromatography)-ICP-MS, and centrifugal ultrafiltration followed by ICP-MS) to quantify metallodrug–protein adducts formed in serum [169]. The metallodrugs used were two Pt(IV) drugs, KP2156 (trans-bis(2-maleimideethylcarbamato)-dihydroxido(*1R*,*2R*-diamminocyclohexane)-oxalatoplatinum(IV)) and KP2157 (trans-bis(2-succinimideethylcarbamato)dihydroxido(*1R*,*2R*-diamminocyclohexane)-oxalatoplatinum(IV)), and the biological fluid was FCS. Only albumin complexes and LMW species were resolved. On balance, Ultra-HPLC-ICP-MS using the latest column material technology (Acquity UPLC Protein BEH SEC, size range 1–80 kDa; 50 mM NH_4_Ac, pH 6.0) provided the best performance and was deemed suitable for high-throughput screening for preclinical studies.

### 3.2. Ruthenium Complexes

Following the success of Pt metallodrugs, attention has turned to the development and metalloproteomic study of ruthenium (Ru) agents which can overcome clinical Pt resistance. A review of metallomic studies of Ru-based metallodrugs is available [170].

The Ultra-HPLC-ICP-MS methodology mentioned above was also used to assess the serum-binding properties of the Ru-based anticancer drug plecstatin-1 and its Os(II) analogue [171]. Both metals were detected in fractions containing transferrin, albumin, and immunoglobulins.

The Ru-based drug KP1019 (Indazolium trans-[tetrachloridobis(1*H*-indazole)ruthenate(III)]) has been investigated extensively using metalloproteomics as part of its preclinical evaluation. The following studies illustrate various stages, from interactions with plasma proteins to intracellular fate and binding to protein targets. Plasma-binding properties were studied using SEC-AEX-ICP-MS (Tosoh Bioscience BioAssist G3SWX; 150 mM NaCl and 20 mM Tris-HCl, pH 7.4, and Tosoh TSK-DEAE NPR; 150 mM NaCl and 20 mM Tris-HCl, pH 10, 1–0.5 M NaCl gradient, respectively) [172] and CE-ICP-MS (50 mM formic acid, pH 2.7) [173]. In both cases, Ru was found in albumin- and transferrin-containing fractions, with a preference for albumin. Based on the recently contested [174] hypothesis that KP1019 entered cells bound to transferrin through its receptor, the fate of transferrin-bound KP1019 was studied using CE-ICP-MS [175]. The dissociation of the Ru-transferrin complex in the presence of cancer cell cytosol components (in particular, glutathione and ascorbate) was demonstrated; the loss of Fe could also be observed. With the aim of protein target identification, cytosolic Ru speciation in human HT-29 colon carcinoma cells treated with KP1019 was studied by shotgun proteomics [176]. The observation of a good number of ruthenated peptides, derived from several proteins including some related to cancer (e.g., p53, BRCA1 and 2) illustrates the persistence of Ru-protein complexes even after denaturation and digestion. The same study also employed SEC-ICP-MS (GE Healthcare Superdex 200 10/300 GL; 10 mM NH_4_Ac, pH 6.0) and CE-ICP-MS (10 mM phosphate buffer containing 4 mM sodium chloride, pH 6.0) to monitor overall speciation. Speciation profiles changed in a time-dependent manner, highlighting the opportunity to monitor the fate of metallodrugs over time.

RAPTA-type drugs have also been the subject of metalloproteomic studies (RAPTA = ruthenium-arene pta, (where pta = 1,3,5-triaza-7-phosphaadamantane)). The extracellular speciation of RAPTA-C [Ru(η^6^-*p*-cymene)Cl_2_(pta)], determined using CE-ICP-MS, has been contrasted with its cellular accumulation profile. Compared to cisplatin, which was found to readily form adducts with both transferrin and albumin, RAPTA-C appeared to react exclusively with albumin [177]. In contrast, earlier SEC-ICP-MS (Superdex 200 HR 10/30, 100 mM NH_4_Ac, and 20 mM Tris-saline, pH 7.2) studies of the structurally similar Ru arene complex, RAPTA-T [Ru(η^6^-toluene)Cl_2_(pta)], indicated a strong preference for Ru-transferrin adduct formation, with higher affinity observed for holo-transferrin than the apoprotein, which was further characterised using ESI-MS [178].

Michelucci and co-workers scrutinised factors throughout the analytical process during bottom-up proteomics that could influence or disrupt metallodrug–protein adduct stability [179]. The workflow involved FAST and nano-HPLC-nano-ESI-LTQ-Orbitrap MS. Adducts formed between cytochrome c and either RAPTA-C (Ru(II)) or cisplatin (Pt(II)) were used as case studies [179]. Critical factors identified included choice of initial sample buffer, denaturation with urea, reduction with dithiothreitol, alkylation with iodoacetamide, sample persistence in the loading and elution mobile phases, the nano-ESI process, transfer of the adduct through an ion transfer tube and a tube lens, and the collision-induced dissociation (CID) process in the ion trap. While cisplatin adducts were found to be highly stable throughout, RAPTA-C adducts were unstable in NH_4_HCO_3_ buffer.

Activation mechanisms and binding preferences of three Ru-based complexes (RAPTA-C, NKP-1339 ([RuCl_4_(HInd)_2_], where Hind = indazole), and RM175 ([(η^6^-biphenyl)RuCl(1,2-ethylenediamine)]PF_6_) were investigated using capillary zone electrophoresis (CZE; 25 mM NH_4_HCO_3_, pH 7.9) coupled to ESI-MS and ICP-MS [180]. The metallodrugs were incubated with a mixture of protein (ubiquitin as model) and DNA (5′-dATTGGCAC-3′ as model). By tracking ^102^Ru^+^, ^48^SO^+^ (protein), ^47^PO^+^, or ^31^P^+^ (DNA) through ICP-MS, the approach allowed for the simultaneous identification and quantitation of LMW species as well as adducts with protein and DNA, in a time-dependent manner. Using tandem-ESI-MS, it was also possible to identify activation products and to locate binding sites at single-amino acid and single-nucleotide resolution. Interestingly, the three metallodrugs showed rather different binding preferences: RAPTA-C preferred methionine, adenine, and cytosine, whilst RM175 preferred guanine, with only very little protein binding observed. Remarkably, the mild conditions also allowed for the direct observation of a Ru(II)-DNA adduct, resulting from reaction with NKP-1339.

In summary, as suggested by water exchange rates (Figure 1), biomolecular complexes formed with Ru(II) and Ru(III) tend to be less robust than those with Pt. Nonetheless, with careful control of conditions, at least some Ru complexes have been shown to be amenable to bottom-up proteomics.

## 4. Gold Complexes

Drugs such as aurothioglucose, aurothiomalate, and auranofin are based on Au(I) and, owing to their anti-inflammatory activity, are in current clinical use to combat arthritis. There is also vigorous research activity on Au(I)- and Au(III)-based anticancer agents [181,182,183]. Au(I) and Au(III) complexes are not kinetically inert and, indeed, their fast ligand exchange kinetics as well as their high likelihood to undergo reduction (especially in the reducing environment of the cytosols) has proven to be a bottleneck for bringing further gold-based drugs into the clinic. However, like the complexes considered in Section 3, Au(I)/Au(III)-based drugs also often only form only one bond with a protein, and the bonds formed tend to be strong and cannot be dissociated as easily as those for most 3d row metal ions (for example, by exposure to a low pH). Hence, in terms of sample handling, some gold-protein complexes may be similarly as robust as their more inert neighbours to the left in the periodic table, so long as exposure to reductants and competing ligands can be avoided. Indeed, one of the earliest studies harnessing the power of coupling separation techniques and elemental analysis to understand the fate and mode of action of metallodrugs pertains to auranofin interacting with serum proteins [184]. More recently, the speciation of gold resulting from exposure of gold nanoparticles (5–50 nm size) to serum proteins in real serum conditions was investigated using capillary electrophoresis (CE) (40 mM HEPES, pH 7.4 at 37 °C) coupled to ICP-MS [185]. Au speciation was dominated by albumin, with transferrin playing a secondary role.

Further studies [183] have focused on reaction products with isolated model proteins rather than complex mixtures. For example, complexes resulting from reactions of either albumin or carbonic anhydrase with a panel of Au(III) drugs were stable enough to be characterised using ESI-MS in the presence of 0.1% formic acid, although the reduction of Au(III) to either Au(I) or Au(0) was observed in some cases [186]. The selective targeting of protein thiols by auranofin was also shown by ESI-MS [187].

## 5. Conclusions

Undoubtedly, since its inception over two decades ago, metalloproteomics has gained traction in a range of areas within the “metals in biology” and “metals in medicine” fields. This review has revealed that much progress has been made with respect to identifying targets for xenobiotic metal ions (and their complexes), both in terms of studying mechanisms of toxicity (including those of labile metal ions) and mode of action of metallodrugs. The success in the latter area is partially owed to the relative inertness and/or thermodynamic stability of metal-ligand bonds for these metal ions. It may be predicted that applications in these fields will continue to provide invaluable insights to understand and harness metal–protein interactions, especially in the context of anticancer therapy. In contrast, the elucidation of entire native metalloproteomes has proven elusive and will likely remain so for the foreseeable future, for the reasons set out in Section 2. Nonetheless, many of the studies included have demonstrated that with careful experimental design that reflects the general considerations and mandates laid out in Section 2.1, metalloproteomic studies aimed at major binding partners for essential metal ions are feasible and enhance our understanding of important biological processes. Finally, it may be stressed that the analytical approaches highlighted here are rarely ever used in isolation but are typically part of more comprehensive studies that may include physiology, cell biology, structural biology including modelling, and bioinformatics.

## Figures and Tables

**Table 1:** Challenges and consequences for experimental design for metalloproteomics of labile metal–protein complexes.

Challenge	Applies to	Mandates to
Metal sites require folded proteins	Intra- and extracellular metalloproteomes	Work under near-physiological conditions
Protons compete for metal sites	Intra- and extracellular metalloproteomes	Avoid low pH
The inside of cells is a reducing environment, and exposure to oxygen can lead to oxidation of metals and/or certain ligands (e.g., thiolates)	Intracellular Metalloproteomes	Work under inert atmosphere or add reducing agent
Removal of intracellular barriers can lead to metal redistribution	Intracellular metalloproteomes	Carry out sub-cellular fractionation

## Abbreviations

2D-GE	2-Dimensional Gel Electrophoresis
Ac	Acetate
AEX	Anion Exchange Chromatography
BSA	Bovine Serum Albumin
CE	Capillary Electrophoresis
CFS	Cerebrospinal fluid
DDM	n-Dodecyl-β-D-maltoside
ESI-MS	Electrospray Ionisation Mass Spectrometry
EDTA	2,2′,2′′,2′′′-(Ethane-1,2-diyldinitrilo)tetraacetic acid
FBS/FCS	Fetal Bovine Serum/Fetal Calf Serum
FPLC	Fast Protein Liquid Chromatography
GE	Gel Electrophoresis
HEPES	2-[4-(2-Hydroxyethyl)piperazin-1-yl]ethane-1-sulfonic acid
HMW	High-molecular-weight
HPLC	High-Performance Liquid Chromatography
HSA	Human Serum Albumin
ICP-OES	Inductively-Coupled Plasma Optical Emission Spectroscopy
ICP-MS	Inductively-Coupled Plasma Mass Spectrometry
IDA	Isotope Dilution Analysis
IDMS	Isotope Dilution Mass Spectrometry
IEF	Isolectric Focusing
LA	Laser Ablation
LMW	Low-molecular-weight
MALDI	Matrix-Assisted Laser Desorption Ionisation
MOPS	3-(Morpholin-4-yl)propane-1-sulfonic acid
PAGE	Polyacrylamide Gel Electrophoresis
PBS	Phosphate-Buffered Saline
PEEK	Polyether ether ketone
SAX	Strong Anion Exchange
SDS	Sodium dodecyl sulfate
SEC	Size-Exclusion Chromatography
TCEP	3,3′,3′′-Phosphanetriyltripropanoic acid
Tris	2-Amino-2-(hydroxymethyl)propane-1,3-diol
TPEN	*N*^1^,*N*^1^,*N*^2^,*N*^2^-Tetrakis[(pyridin-2-yl)methyl]ethane-1,2-diamine
